# Advances in the mTOR signaling pathway and its inhibitor rapamycin in epilepsy

**DOI:** 10.1002/brb3.2995

**Published:** 2023-05-23

**Authors:** Wei Zhao, Cong Xie, Xu Zhang, Ju Liu, Jinzhi Liu, Zhangyong Xia

**Affiliations:** ^1^ Department of Gerontology The First Affiliated Hospital of Shandong First Medical University & Shandong Provincial Qianfoshan Hospital Jinan China; ^2^ Laboratory of Microvascular Medicine Medical Research Center Shandong Provincial Qianfoshan Hospital, Shandong University Jinan China; ^3^ Department of Neurology Liaocheng People's Hospital and Liaocheng Clinical School of Shandong First Medical University Liaocheng China; ^4^ Department of Gerontology Cheeloo College of Medicine Shandong Provincial Qianfoshan Hospital, Shandong University Jinan China; ^5^ Department of Geriatric Neurology The First Affiliated Hospital of Shandong First Medical University & Shandong Provincial Qianfoshan Hospital Jinan China; ^6^ Department of Neurology Cheeloo College of Medicine Liaocheng People's Hospital, Shandong University Jinan China

**Keywords:** epilepsy, mTOR, rapamycin, signaling pathway

## Abstract

**Introduction:**

Epilepsy is one of the most common and serious brain syndromes and has adverse consequences on a patient's neurobiological, cognitive, psychological, and social wellbeing, thereby threatening their quality of life. Some patients with epilepsy experience poor treatment effects due to the unclear pathophysiological mechanisms of the syndrome. Dysregulation of the mammalian target of the rapamycin (mTOR) pathway is thought to play an important role in the onset and progression of some epilepsies.

**Methods:**

This review summarizes the role of the mTOR signaling pathway in the pathogenesis of epilepsy and the prospects for the use of mTOR inhibitors.

**Results:**

The mTOR pathway functions as a vital mediator in epilepsy development through diverse mechanisms, indicating that the it has great potential as an effective target for epilepsy therapy. The excessive activation of mTOR signaling pathway leads to structural changes in neurons, inhibits autophagy, exacerbates neuron damage, affects mossy fiber sprouting, enhances neuronal excitability, increases neuroinflammation, and is closely associated with tau upregulation in epilepsy. A growing number of studies have demonstrated that mTOR inhibitors exhibit significant antiepileptic effects in both clinical applications and animal models. Specifically, rapamycin, a specific inhibitor of TOR, reduces the intensity and frequency of seizures. Clinical studies in patients with tuberous sclerosis complex have shown that rapamycin has the function of reducing seizures and improving this disease. Everolimus, a chemically modified derivative of rapamycin, has been approved as an added treatment to other antiepileptic medicines. Further explorations are needed to evaluate the therapeutic efficacy and application value of mTOR inhibitors in epilepsy.

**Conclusions:**

Targeting the mTOR signaling pathway provides a promising prospect for the treatment of epilepsy.

## Graphical abstract

The regulation of the mTOR pathway provides a potential strategy for the treatment and prevention of epilepsy.

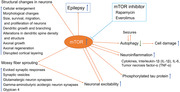

## 1 INTRODUCTION

Epilepsy is a disabling chronic neurological syndrome characterized by recurrent epileptic seizures attributable to multiple genetic and acquired causes. There are 50 million newly diagnosed epilepsy patients and 125,000 deaths from epilepsy worldwide every year (Singh & Sander, 2020). Drug therapy is the most important treatment for epilepsy, with the ability to prevent seizures in two‐thirds of patients. As a supplement to drug therapy, surgery is the most effective treatment for drug‐resistant focal epilepsy. It is well documented that the series of adverse effects induced by epilepsy on a patient's neurobiological, cognitive, psychological, and social wellbeing threaten their quality of life. As evidenced by the Global Burden of Epilepsy Report, epilepsy is a serious impediment to public health (Singh & Sander, 2020). The imbalance of excitation and inhibition in the central nervous system induces recurrent seizures, reflecting the underlying pathological mechanism of the disease (Beghi, 2020). A deeper understanding of the pathological mechanism of epilepsy and the improvement of biological research techniques have strongly supported the research for new strategies to balance the nervous system and reduce seizures. At present, the exploration of the key regulatory signaling pathways involved in epilepsy and the therapeutic drugs that targer them is of great significance for the prevention and treatment of this disease.

As the first‐line treatment for epilepsy, drug therapy improves the excitatory/inhibitory imbalance of the brain after seizures by targeting ion channels or neuronal transmission. Although drug treatment can suppress seizure frequency and ictal discharges, it has a nonobvious effect on reducing interictal epileptiform discharge (Hodges & Lugo, 2020). More significantly, existing treatments are ineffective for about one‐third of patients suffering from refractory epilepsy (Palleria et al., 2015; Potschka & Brodie, 2012). Moreover, even if seizures are controlled by drugs, those currently available are generally considered for symptomatic treatment, which can only inhibit the terminal stage of seizures. In other words, although these drugs can control seizures, there is little evidence that they have a disease‐modulating ability to prevent or slow the development of epilepsy (Temkin, 2001). Therefore, a new generation of drugs that address the signaling pathways involved in epileptogenesis is urgently needed to prevent seizures.

The serine‐threonine kinase mTOR, is a key porint in the important eukaryotic signaling network that coordinates cell growth with environmental conditions and acts as a core regulator for many other physiological functions. In the nervous system, mTOR plays an important role in synaptic plasticity, brain development, and neuronal survival (Lee, 2015; Limanaqi et al., 2020; Pagani et al., 2021). Many animal experiments have demonstrated that activation of the mTOR pathway is closely related to the occurrence of epilepsy (Lee, 2015; Russo et al., 2012, 2013; Leo et al., 2016). The mTOR signaling is involved in highly epileptogenic diseases, including tuberous sclerosis complex (TSC), and it is a reasonable target for antiepileptic intervention (Salussolia et al., 2019). The mTOR inhibitors can prevent epilepsy and reduce potential brain abnormalities. Preliminary clinical studies in TSC patients have shown that mTOR inhibitors have the function of reducing seizures and improving this disease. For instance, rapamycin (sirolimus), a specific inhibitor of TOR, reduces the intensity and frequency of seizures (Marsan & Baulac, 2018; Stefanidou et al., 2017), and represents a new therapeutic approach for refractory epilepsy. The function of mTOR and its underlying regulatory mechanisms have only been revealed in recent years, and further elucidation requires more in‐depth exploration. Accordingly, this review focuses on the latest findings of the mTOR signaling pathway and rapamycin in the treatment of epilepsy.

## 2 MTOR SIGNALING PATHWAY

MTOR, a highly conserved serine/threonine protein kinase, is widely expressed in eukaryotic cells and serves as an atypical membrane of the phosphatidylinositol 3‐kinase (PI3K) related kinase family (Lipton & Sahin, 2014). The mTOR signaling pathway is essential for the proper functioning of physiological processes in mammals. When performing biological functions, the mTOR protein binds to several regulatory proteins to form two protein complexes with different functions, which are referred to as mTOR complex 1 (mTORC1) and mTOR complex 2 (mTORC2) (Chong et al., 2012). Under normal physiological conditions, mTORC1 regulates cell growth, proliferation, and survival, mainly by controlling ribosome biogenesis and protein translation. The GTP‐binding protein Rheb can result in the suppression of mTORC1, while Rheb is inhibited by a complex formed by hamartin and tuberin proteins, which are encoded by the TSC1 and TSC2 genes. In the state of excess energy, the active PI3K/Akt pathway causes activation of the mTOR signaling pathway under the stimulation of several growth factors (e.g., insulin), leading to increased cell growth and metabolism. Similarly, mTOR can be activated by the DEPDC5 pathway, which is triggered by high levels of amino acids (Samanta, 2022). In contrast, activation of the liver kinase B1 and its downstream target AMP‐activated protein kinase (LKB1‐AMPK axis) inhibits mTORC1 under the energy deficiency, which leads to the limitation of cell growth (Beirowski, 2019). Unlike the extensive elucidation of mTORC1 functions, little is known about the functions and effector substrates of mTORC2 (Fu & Hall, 2020). According to existing research, mTORC2 is involved in cell survival processes, such as cell differentiation, metabolism, autophagy, proliferation (Ballesteros‐Alvarez & Andersen, 2021; Bernard et al., 2020; Guri et al., 2017), and recombination of the actin cytoskeleton (Xie et al., 2018). Ras and its downstream gene PI3K seem to be inducers of mTORC2 activation (Smith et al., 2020). The effector substrates of mTORC2 have been suggested to be PKC, Akt, and newly identified molecules (Baffi et al., 2021; Torti et al., 2020). Likewise, some upstream regulators of mTORC2 have recently been discovered (Bhat et al., 2022; Hau et al., 2017; Kazyken et al., 2019; Wrobel et al., 2020). However, these mTORC2 regulatory molecules need further verification. Generally, the complex signaling mechanisms and feedback connections of mTORC1 and mTORC2 contribute to the ability of cells to sensitively regulate various cellular processes (including metabolism, proliferation, apoptosis, and autophagy) in response to different environments. The dysfunction of mTORC1 and mTORC2 has been recognized in the pathological processes of many diseases, such as cancer and diabetes.

In the nervous system, mTOR is involved in neuronal development and the normal function of mature neurons (Switon et al., 2017). However, mTORC1 and mTORC2 also have different impacts on neurons. Inhibition of mTORC1 mainly affects the structure and function of dopamine neurons, promotes dendritic and axonal atrophy, increases neuronal excitability, and inhibits dopamine release, whereas suppressing mTORC2 alters the output of dopamine neurons (Kosillo et al., 2022). Furthermore, mTORC2 performs an important role in neuronal morphology and synaptic function by regulating the rearrangement of the actin cytoskeleton (Angliker & Ruegg, 2013). Both mTORC1 and mTORC2 mediate neuronal signaling and excitability, such as dendritic growth and morphology, synaptic transmission and plasticity, neurogenesis, and neural network activity (Kosillo et al., 2022), which are essential for normal cortical development, cognition, and behavior.

Dysregulation of the mTOR pathway has been correlated with a variety of neurological disorders, thereby providing a new therapeutic strategy for restoring neurological function. The imbalance of mTORC1 and mTORC2, which is involved in promoting neuronal death, has been recognized in a variety of neurodegenerative diseases, such as Parkinson's disease, Huntington's disease, frontotemporal dementia, and amyotrophic lateral sclerosis (Querfurth & Lee, 2021). Studies show that inhibition of mTOR can alleviate the pathological features and progression of neurodegenerative diseases both in vitro and in vivo and may even affect neuronal development (Granatiero et al., 2021) (Kim et al., 2021; Gugliandolo et al., 2020; Pupyshev et al., 2021; Williams et al., 2008).

## 3 THE ROLE OF THE MTOR SIGNALING PATHWAY IN EPILEPSY

**FIGURE 1:**
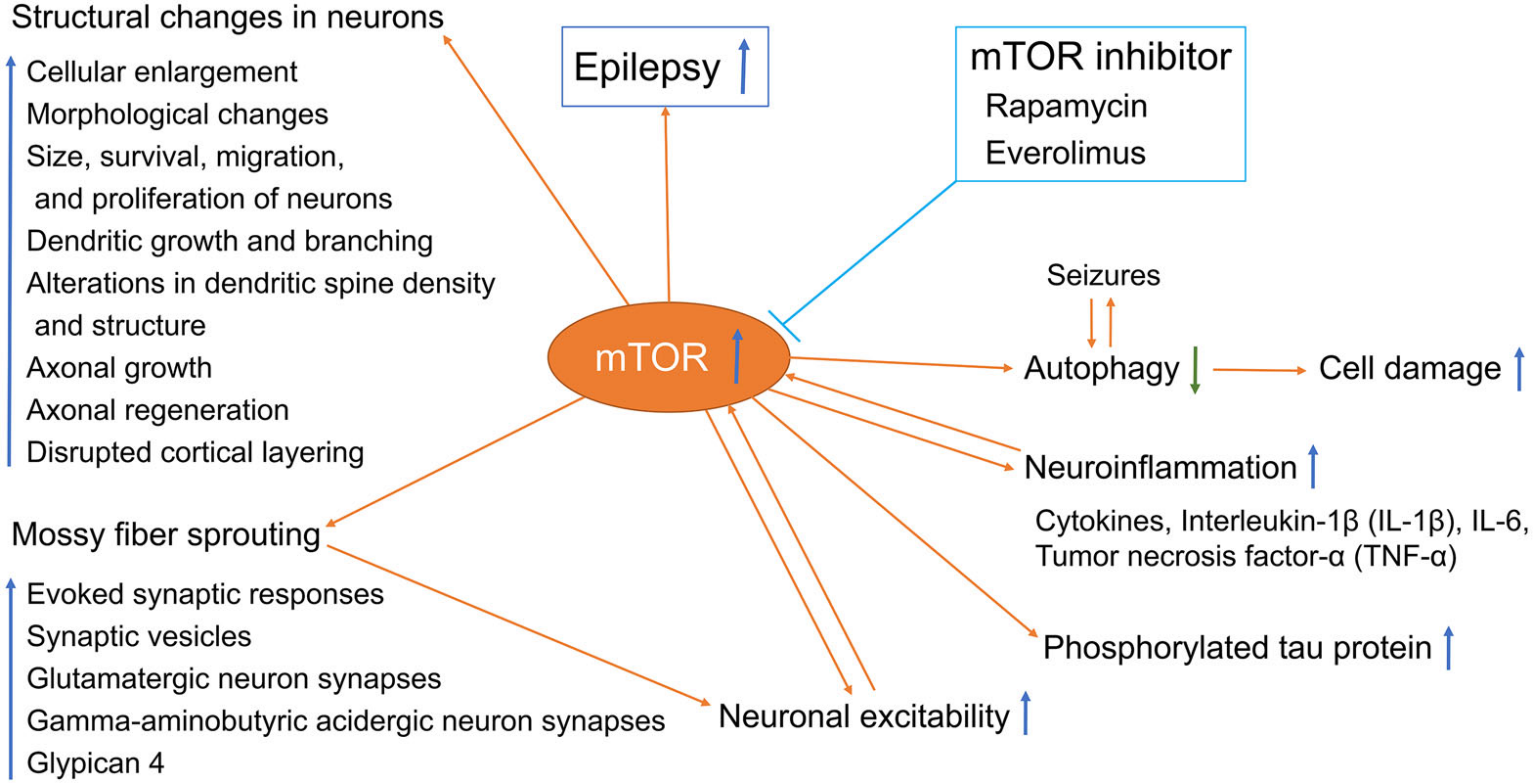
mTOR dysfunction in epilepsy. MTOR is overactivated and induces the pathogenesis of epilepsy by disrupting the formation of neural loops and altering existing neural networks. Multiple mechanisms of mTOR in epilepsy have been proposed. Cellular events associated with epilepsy pathologies, such as neuron structure, autophagy‐correlated neuron damage, neuronal excitability, and neuroinflammation, are recognized as responders to the mTOR pathway.

Although it has been observed for many years that mTOR inhibition contributes to antiepileptogenic therapy, the regulatory mechanism of mTOR in seizure‐induced neurological changes is not well known. Interestingly, emerging studies have demonstrated that overactivated mTOR induces the pathogenesis of epilepsy by disrupting the formation of neural circuits and altering existing neural networks (Mohammadi et al., 2022; Nguyen et al., 2022). Thus, whether epilepsy is a direct consequence of mTORC1 overactivation or an inevitable result of disrupted neural networks caused by abnormal cortical structures has become the focus of many discussions. In any case, mTOR is considered a collaborative molecule for epilepsy pathogenic factors (Iffland et al., 2022; Zeng et al., 2022). In basic research on epilepsy treatment, the mTOR signaling pathway is recognized as a key regulatory hub for several drugs that can inhibit drug‐induced seizures in rat models of epilepsy (El‐Sayed et al., 2021; Mazumder et al., 2019). The exact mechanism by which the overactivation of mTOR signaling leads to neuronal hyperexcitation and seizures remains to be fully elaborated. Even so, recent studies have revealed multiple ways in which the mTOR pathway triggers or exacerbates epilepsy (Figure 1).

### 3.1 Structural changes in neurons caused by excessive activation of the mTOR signaling pathway as the key to epilepsy

Phosphatase and tensin homolog (PTEN), a well‐known cancer suppressor, can negatively regulate the mTOR pathway. In hippocampal granule cells from PTEN deletion mice, low knockout has been shown to lead to focal seizures, while high knockout results in generalized seizures, indicating that mTOR signaling is closely related to neuronal loss and generalized seizures (LaSarge et al., 2021). The mTOR signaling pathway plays a key role in nerve growth and development, such as affecting the size, survival, migration, and proliferation of neurons, controlling dendritic growth and branching, and regulating axonal growth. For example, mTOR is activated after neuronal injury and enhanced axonal growth in mouse dorsal root ganglion neurons (DRGNs) (Abe et al., 2010). Furthermore, activation of mTOR by repressing PTEN or TSC1 can potently promote axonal regeneration in the central nervous system, with a weak regenerative capacity (Wei et al., 2019). Mutations in active regulatory genes (TSC1, TSC2, AKT3, and DEPDC5) has been shown to cause activation of the mTOR signaling cascade, resulting in a series of pathological events associated with increased excitability and decreased seizure threshold, including celluar enlargement and morphological changes, dendritic morphology changes, dendritic spine density and structure changes, axonal regeneration, and cortical layering disorder due to altered neuronal movement (Crino, 2015). The mTOR cascade activation may be an important cause of TSC‐related epilepsy, a form of epilepsy caused by malformations of cortical development (MCDs) (Curatolo, 2020). In TSC1‐deleted mice, epilepsy occurs within a short time window (8‐12d) of TS and is accompanied by strong activation of the mTORC1 pathway, showing a dose‐dependent response to the mTORC1 inhibitor rapamycin. In addition, both RHEB1 deletion and rapamycin treatment have been found to completely prevent the development and lethality of epilepsy (Koene et al., 2019). This evidence conceivably suggests that excessive activation of the mTOR signaling pathway is a key factor in epileptogenesis.

### 3.2 Excessive activation of the mTOR signaling pathway inhibits autophagy and exacerbates neuron damage

Autophagy is a conserved mechanism that maintains the cellular homeostasis involved in cell survival, metabolism, and growth by preventing the accumulation of abnormal proteins and removing damaged organelles. Seizures can trigger signaling pathways related to apoptosis, cellular or mitochondrial metabolism, and autophagy, and vice versa. In particular, these pathways play an important role in epilepsy‐induced neuronal loss. Autophagy occurs prior to apoptosis, indicating the significance of autophagy in the loss of brain neurons in epilepsy (Islin et al., 2018). The activation/inhibition transition of autophagy is a complex process regulated by multiple signaling pathways. Increasing evidence suggests that mTOR is a crucial mediator in autophagy. Under conditons of nutrient or amino acid depletion and decreased ATP or oxygen levels, the response of mTOR signaling to cellular stress is weakened, thereby initiating autophagy to alleviate cellular damage (Crino, 2016). The regulatory effect of mTOR on neuronal autophagy has also been validated in animal and cellular models of epilepsy. In a rat model of pilocarpine‐induced epilepsy, activation of the mTOR signaling pathway inhibits neuronal autophagy and avoids neuronal apoptosis, which can prevent acute epilepsy (Liu et al., 2022). Furthermore, autophagy dysfunction exists in epilepsy (Zhu et al., 2022), and the impairment of mTOR‐dependent autophagy has been found to be related to the mechanism by which mTOR overactivation promotes epilepsy (Limanaqi et al., 2020). Rapamycin induces early activation of the autophagy cascade via blockade of mTOR, while activation of mTOR inhibits the formation of autophagosomes (Crino, 2016). By analyzing the differentially expressed genes (DEGs) in epilepsy rat models with or without electroacupuncture treatment, the functional enrichment of DEGs was found mainly in the mTOR signaling pathway and autophagy (Gao et al., 2022). Thus, there is a reliable correlation between mTOR‐dependent autophagy and seizure onset and seizure‐induced neuronal damage. Accordingly, targeting the mTOR signaling pathway has been found to regulate autophagy in the hippocampal neurons of status epilepticus (SE) rats (Wu et al., 2020).

### 3.3 MTOR signaling pathway affects mossy fiber sprouting

The pathological feature of temporal lobe epilepsy (TLE) is mossy fiber sprouting in the hippocampus, which results from abnormal axon guidance and synapse formation (Ma et al., 2022). The importance of mTOR pathway targets in mossy fiber sprouting has been confirmed (Tang et al., 2012). Mossy fiber sprouting represents synaptic reorganization, leading to the formation of abnormal recurrent excitatory circuits and inputs. By regulating synaptic function and plasticity, the mTOR pathway affects neuronal excitability and higher physiological functions, such as cognition, feeding, and circadian rhythm control. In a PTEN knockout mouse model, the activation of mTOR signaling increases the evoked synaptic responses, the number of synaptic vesicles, and the number of synapses in glutamatergic and gamma‐aminobutyric acid‐ergic neurons (Weston et al., 2012). Rapamycin can prevent these changes and reduce synaptic transmission in wild‐type glutamatergic neurons. In mice with pilocarpine‐induced SE, mTOR signaling has been found to be necessary for the regulation of mossy fiber sprouting by glypican 4, an important protein for axon guidance and excitatory synapse formation (Ma et al., 2022).

### 3.4 Activation of mTOR signaling pathway enhances neuronal excitability

Enhanced mTOR signaling may be a critical activation step in epileptogenesis, even in the absence of obvious neuropathological changes. In a study with TSC1 knockout rats, severe epilepsy occured in adult rats without obvious changes in brain structure, suggesting that enhanced mTOR signaling may contribute to epileptogenesis through unknown mechanisms rather than structural changes in brain tissues (Abs et al., 2013). In addition, rapamycin treatment has been found to decrease TORC1 activity and eliminate seizure symptoms in these rats (Abs et al., 2013). In a mouse model of mesial‐temporal lobe epilepsy (MTLE), the mTOR signaling pathway is activated by increased neuronal excitability in the dispersed granule cell layer (GCL) of the dentate gyrus. While long‐term administration of rapamycin inhibits the p‐S6 expression and mossy fiber germination, it dose not affect cell loss or paroxysmal discharge in the hippocampus (Shima et al., 2015). The inhibition of the mTOR signaling pathway does not improve MTLE (Shima et al., 2015).

### 3.5 Dysregulation of mTOR signaling pathway increases neuroinflammation

Increasing evidence suggests that activation of neuroimmune cells (e.g., microglia and astrocytes) and peripheral immune cells, as well as accompanying inflammatory mediators, are both causes and consequences of epilepsy pathogenesis. Of note, inflammatory mediators, particularly interleukin‐1β (IL‐1β), IL‐6, and tumor necrosis factor‐α (TNF‐α), can be detected in surgically resected brain tissue samples from patients with intractable epilepsy (Hodges & Lugo, 2020; Soltani Khaboushan et al., 2022). Furthermore, animal experiments demonstrate that seizures can induce brain inflammation and that recurrent seizures perpetuate chronic inflammation (Erisken et al., 2022; Wu et al., 2018). Maternal immune activation, autoimmune neurodegenerative disease, and preexisting brain inflammation can increase susceptibility to seizures (Chen et al., 2021; Glass et al., 2009; Karabulut et al., 2022), which is associated with altered neuronal excitability and enhanced neuropathology that induces epilepsy. Although the bidirectional mechanism of the interaction between epilepsy and neuroinflammation is not fully understood (Bakhtiar & Selwyn, 1988), the correlation between epilepsy and neuroinflammation is beyond doubt.

Emerging evidence suggests that mTOR acts as a bridge between neuroinflammation and epilepsy. In TLE, mTOR modulates cytokine production in peripheral blood leukocytes (Vieira et al., 2021). Xiao et al. (2015) found that IL‐1β‐induced inflammation and epileptic seizure were suppressed by inhibition of the mTOR signaling pathway in a rat model of MTLE. Drugs that inhibit mTOR pathway activation have also been found to improve neuronal damage and neuroinflammation in the hippocampus (Park et al., 2022). For instance, rapamycin inhibits the inflammatory response by inhibiting microglia activation. Clinically, when mTOR is inhibited, cytokines produced by immune cells in TLE patients are different from those produced by immune cells in nonepilepsy patients, which is associated with changes in the activity and reactivity of PI3K, mTOR, and GSK‐3, indicating that mTOR may be involved in the inflammation and pathogenesis of epilepsy (Vieira et al., 2021). Moreover, increased IL‐1β expression has been show to be strongly correlated with the overactivation of the PI3K/AKT/mTOR pathway. Specifically, IL‐1β can directly activate PI3K by facilitating the phosphorylation of AKT, thereby leading to the upregulation of mTOR (Xiao et al., 2016).

### 3.6 MTOR activation is strongly associated with tau upregulation

Tau, a microtubule‐associated protein, is upregulated in neurons via abnormal hyperphosphorylation in several adult neurodegenerative diseases, such as Alzheimer's disease, Parkinson's disease, and frontotemporal lobe degeneration; this upregulation is also a leading contributor to dementia in adults (Kovacs, 2015). In the early stage of cell maturation, muscle tube disorders caused by abnormal expression of tau have been shown to interfere with the programmed growth and morphology of neurons, which is the main factor in leading to altered cortical architecture and abnormal neuronal/glial morphology (Mühlebner et al., 2019). Some structural‐developmental brain malformations, including symptomatic epilepsy (PMSE) syndrome, focal cortical dysplasias (FCDs), hemimegalencephaly (HME), gangliogliomas resulting from malignant transformation of cortical dysplasia, and dysembryonplastic neuroepithelial tumors (DNETs), are classified as “mTORopathies” due to their common characteristics of disrupting the mTOR pathway (Blümcke et al., 2021; Moloney et al., 2021; Wong, 2013; Xu et al., 2019). Despite differences in their histological structure, they are clinically featured as intractable epilepsy, indicating that mTOR plays a role in epileptogenesis. The occurrence of these mTORopathies is correlated with dysmorphic neurons and incomplete cellular maturation. More importantly, abnormal phosphorylation of tau is enhanced in these diseases, suggesting that their common pathogenesis may involve microtubule function in the early stages of neuronal differentiation (Sarnat & Flores‐Sarnat, 2015). Furthermore, neurotoxic phosphorylated tau could be responsible for the epileptogenesis of the indicated mTORopathies in infancy (Sarnat & Flores‐Sarnat, 2015). In general, mTOR activation is strongly associated with the upregulation of tau in epileptogenic brain malformations.

## 4 MTOR INHIBITOR APPLICATION IN TREATING EPILEPSY

### 4.1 Rapamycin

Rapamycin is a macrolide antibiotic generated by streptomyces hygroscopicus, which was first isolated from soil samples collected by a Canadian expedition in 1964 on the South Pacific island of Rapa Nui (Laplante & Sabatini, 2012). This compound is named after the discovery site (clinically known as sirolimus). Subsequently, it has been demonstrated that rapamycin inhibits the signal transduction pathways required for cell growth and proliferation by forming a functional complex with the peptidyl‐proline‐isomerase FK‐506 binding protein 12 (FKBP12) (Jayaraman & Marks, 1993). Since rapamycin has been identified as a direct target of mTOR, some evidence for its role in antiepilepsy has been accumulated (Ryskalin et al., 2018).

Rapamycin specifically inhibits the mTOR activity but exhibits different effects on the two protein complexes. More specifically, mTORC1 can be acutely inhibited due to its higher sensitivity, while long‐term treatment is needed to suppress mTORC2 because of its drug resistance (Chong et al., 2012). Rapamycin binds to mTORC1 at the C‐terminus by interacting with the immunoaffinity protein FKBP12, which is a nonspecific mTOR‐interacting protein that binds to rapamycin only by combining with the mTORC1 complex (Lipton & Sahin, 2014). As for mTORC2, rapamycin may disrupt the assembly and integrity of mTORC2 (Chong et al., 2012) by directly interacting with mTOR in the FKBP12 ‐ rapamycin binding (FRB) domain (FRB) (Lipton & Sahin, 2014).

A potential mechanism for the antiepileptic effect of rapamycin is through the immunomodulatory and anti‐inflammatory pathways (Broekaart et al., 2017). A strong inflammatory response occurs in the brain tissue during and after SE. The use of mTOR inhibitors has been shown to attenuate T cell migration and inflammation and reduce SE‐induced neuronal loss, mossy fiber sprouting, and blood‐brain barrier leakage (Bauer & Bien, 2009; Holtman et al., 2013; Shimada et al., 2013). Furthermore, mTOR inhibitors may be conducive to controlling the onset and progression of autoimmune encephalitis and paraneoplastic encephalitis due to their powerful immunosuppressive effects. It has also been hypothesized that mTOR inhibitors have the potential to treat SE syndromes with autoimmune characteristics, such as febrile infection‐related epilepsy syndrome (FIRES) and new onset refractory SE (NORSE). MTOR inhibitors can also be used as an adjuvant therapy to reduce the inflammatory response of encephalitis in the clinic (Crino, 2019). In addition, rapamycin may inhibit the recurrent excitatory circuit in the dentate gyrus by inactivating the mTOR pathway and impeding mossy fiber germination after SE. In a mouse model of TLE, rapamycin decreases the frequency of spontaneous excitatory postsynaptic currents (EPSCs), the amplitude of antidromically evoked EPSCs, epileptiform activity, and mossy fiber sprouting, indicating rapamycin playes an antiepileptic role by inhibiting recurrent excitation circuits of the dentate gyrus (Tang et al., 2012).

In cellular models of epilepsy and TSC, rapamycin has been shown to reduce the frequency and duration of seizures and to have positive effects on cell growth and morphology. Additionally, it has been demonstrated that rapamycin can prevent or ameliorate seizures and prolong survival times in animal models of TSC. To date, clinical studies on the reduction of seizures by rapamycin have focused on TSC patients (Goldstein & Hauptman, 2021). For instance, an open‐label prospective study recruited 52 children suffering from TSC complicated with epilepsy who had received rapamycin treatment (1 mg/m^2^/d) for at least 24 weeks (Zou et al., 2014). Following 24 weeks of rapamycin treatment, the seizure‐free rate of the participants was 25%. Importantly, although rapamycin did not completely eliminate seizures, it reduced seizure frequency (70.27 times/day to 1.94∼2.80 times/day) and lessened the use of antiepileptic drugs (Zou et al., 2014). Sadowski et al. (2022) also conducted an open‐label clinical study to evaluate the safety and efficacy of rapamycin in 32 patients (aged 11 months to 14 years) with drug‐resistant TSC‐related epilepsy. After 6 months of treatment, an obvious reduction in seizure frequency was observed in 18 patients (56.25%). The cumulative dose of rapamycin showed a linear relationship with its therapeutic effect, suggesting that the long‐term use of rapamycin may be an effective treatment for drug‐resistant epilepsy in children with TSC (Sadowski et al., 2022). A related case report described eight cases diagnosed with TSC aged 4–16 years who received rapamycin therapy for 1–5 years because of epileptic seizures and/or accompanying TSC. In continuous follow‐up, the researchers found that rapamycin therapy for TSC was found to have a positive effect on seizures within 1–2 years, but this impact diminished after 2 years (Canpolat et al., 2018). It has also been reported that rapamycin successfully prevented seizures in patients with Pretzel syndrome associated with STRADA mutations (Parker et al., 2013). Of note, rapamycin treatment was administered before seizures in this research, suggesting that pretreatment before seizures may be the key reason for successful treatment (Parker et al., 2013). Based on these facts, it is crucial to determine the appropriate timing, dose, and duration of treatment with an mTOR inhibitor.

### 4.2 Everolimus

Everolimus, a chemically modified derivative of rapamycin, has been approved for the treatment of children and adults with TSC who are unable to undergo subependymal giant‐cell astrocytomas (SEGAs) surgery. A prospective multicenter clinical trial demonstrated the efficacy of everolimus in TSC patients with epilepsy, with 12 of the 20 subjects having a reduction in seizure frequency of more than 50%. The median seizure frequency in 17 patients decreased by 73%, which was beneficial for improving their behavior and quality of life (Krueger et al., 2013). Krueger et al. (2010) also performed an open‐label study evaluating the effect of everolimus on SEGAs in TSC patients and showed that six months of everolimus treatment reduced tumor volume and effectively improved seizure frequency and the quality of life of patients (Krueger et al., 2010). Franz et al. (2015, 2016, 2018) subsequently conducted a long‐term, multi‐dimensional analysis of the clinical application of everolimus and reported the sustained efficacy of everolimus in reducing SEGA volume and seizures, as well as its safety and tolerability for prolonged use.

Overall, the efficacy and safety of mTOR inhibitors are satisfactory, which suggests that this class of drugs may be a beneficial treatment option for refractory epilepsy in patients with TSC. Given the current evidence, it is necessary to investigate the role of mTOR inhibitors in the treatment of children with drug‐refractory, non‐TSC‐related epilepsy.

## 5 CONCLUSIONS

Regulation of the mTOR pathway provides a potential strategy for the treatment and prevention of epilepsy. Everolimus has been approved by the Food and Drug Administration (FDA) for the treatment of seizures in TSC as an adjunct to conventional antiepileptic drugs (AEDs). However, the exact mechanisms of mTOR still need further exploration. The effectiveness of mTOR inhibitors in modulating aberrant mTOR signaling before seizures and ameliorating epilepsy in infants with TSC remains to be elucidated. Generally, despite promising avenues, the application of mTOR inhibitors still has certain limitations. In animal experiments and clinical studies, there is clear evidence that clinical symptoms relapse due to the withdrawal of mTOR inhibitors (Sadowski et al., 2022). In addition, these drugs are associated with potentially serious side effects, including immunosuppression, mucositis, hyperlipidemia, and dysmenorrhea, which may affect long‐term tolerance and adherence. These drugs also have potential off‐target effects. Limited penetration of the blood‐brain barrier may reduce the efficacy in their treatment of neurological diseases (Crino, 2016).

In conclusion, data from animal experiments and clinical studies confirm that mTOR inhibitors show great potential as new drugs for epilepsy treatment. Therefore, addressing the adverse effects of long‐term mTOR inhibition and exploring alternative delivery methods that may alleviate these challenges is important for the future clinical application of these drugs.

## CONFLICT OF INTEREST STATEMENT

The authors declare that there are no conflicts of interest.

### PEER REVIEW

The peer review history for this article is available at https://publons.com/publon/10.1002/brb3.2995.

## Data Availability

Data sharing not applicable to this article as no data sets were generated or analyzed during the current study.
